# Association between electronic device use time and self-rated health in medical students: mediating role of sleep quality and moderating effect of psychological stress

**DOI:** 10.3389/fpubh.2026.1761264

**Published:** 2026-03-12

**Authors:** Xiaolan Mei, Wenjing Pan, Xiaoliang Ding, Xingru Ma, Ruibo Wu, Yuqi Pan, Jiahui Hu, Zhongliang Bai, Xiange Zhang

**Affiliations:** School of Health Care Management, Anhui Medical University, Hefei, China

**Keywords:** e-device use time, medical students, psychological stress, self-rated health, sleep quality

## Abstract

**Background:**

The physical and mental health of medical students not only directly affects academic performance and professional development but also significantly impacts the quality and safety of future healthcare services. Currently, this population faces multiple stressors, including heavy academic workloads, intense clinical training demands, and career-related anxiety. Given these challenges, medical education should prioritize student wellbeing by developing and implementing a comprehensive mechanism to validate the impacts of self-rated health among this specialized population.

**Objective:**

This study was aimed to investigate the relationship and underlying mechanisms between electronic device use time and self-rated health among medical students.

**Methods:**

Convenience sampling was used to survey 699 medical students from universities in Hefei City. The study used self-administered questionnaires to assess electronic device use time, sleep quality, psychological stress, and self-rated health.

**Results:**

The electronic device use time positively predicted the score of self-rated health scale (*β* = 0.149, *p* < 0.001), indicating that longer electronic device use time was associated with poorer self-rated health among medical students. Sleep quality exerted a mediating role between electronic device use time and self-rated health, with a mediating effect size of 0.181 (95% Bootstrap CI = [0.0677, 0.3269]), accounting for 20.03% of the total effect. The first segment of the mediating pathway through which electronic device use time affects medical students’ self-rated health via sleep quality was moderated by psychological stress (*β* = 0.0727, *p* = 0.0550); specifically, higher levels of psychological stress strengthened the negative predictive effect of electronic device use time on sleep quality.

**Conclusion:**

This study elucidates the relationship and mechanisms between electronic device use time and self-rated health among medical students, providing insights to enhance health awareness and promote healthy behaviors.

## 1 Introduction

The health status of college students is not only related to individual growth and development but also exerts a profound impact on the development of the entire country and society. As a special subgroup of college students, medical students represent a key pipeline for the future healthcare workforce and play a critical role in safeguarding population health (1). Therefore, exploring the health behaviors and self-rated health of medical students is of great significance for advancing the Healthy China Initiative. With the advancement of the digital era, electronic media has been deeply integrated into the study and life of medical students, and problems such as mobile phone addiction and screen dependence have gradually emerged. Long-term use of electronic devices, especially excessive use before bedtime, is associated with delayed sleep onset, disrupted sleep patterns, shortened sleep duration, and poor sleep quality (2, 3). Data show that up to 70.32% of college students suffer from sleep problems, such as insufficient sleep and difficulty falling asleep (4), and the overall prevalence of sub-health among medical students reaches 62.2% (5). Additionally, a meta-analysis indicates that sleep duration is associated with self-rated health to varying degrees and linked to increased fatigue and depression rates (6). Based on these observations, Hypothesis 1 was proposed: electronic device usage duration is negatively associated with sleep quality, and sleep quality plays a mediating role between electronic device usage duration and self-rated health.

According to the Knowledge-Attitude-Practice (KAP) theory, appropriate self-assessment of health facilitates the adoption of health-promoting behaviors among college students. Self-rated health (SRH) refers to an individual’s subjective judgment of their own disease burden and a comprehensive expectation of their overall health status (7). Its core value lies in stimulating and guiding individuals to actively perceive pain and discomfort that are difficult to observe through external means, thereby reducing the risk of illness and death (8), and enabling early prevention and timely control of diseases. Existing studies have shown that the SRH status of college students tends to decline with advancing academic year (9) and is significantly associated with sleep duration, personal health literacy, sleep quality, and physical activity level (10). Thus, Hypothesis 2 was put forward: there is a bidirectional relationship between sleep quality and SRH, and sleep quality is significantly positively associated with SRH.

The “China National Mental Health Development Report (2023—2024)” and the 2024 “Mental Health Blue Book” indicate that the prevalence of mental health problems among Chinese adolescents is approximately 29.4%, higher than the global average, making adolescent mental health an urgent social concern. Medical students face challenges such as academic pressure, and clinical training, which may contribute to negative emotional states and psychological difficulties. Psychological stress, also known as mental pressure, is a common psychological state closely associated with physical and mental health as well as functional activities. Chronic or intense psychological stress is a core predisposing factor for academic difficulties and psychological disorders among college students (11). Current academic research on psychological stress mainly focuses on the integration of stress system theory and nursing intervention models to alleviate negative physical and psychological emotional states in patients (12). Existing studies have shown that perceived stress is significantly negatively associated with predicted sleep quality, and individuals under high pressure are prone to negative stress emotions such as anxiety and irritability. Frequent use of social media may impair individuals’ emotional regulation abilities, making it difficult to effectively cope with anxiety and stress (13). With the development of the biopsychosocial medical model, negative emotions are considered traceable to specific psychosocial factors, and high levels of psychological stress may increase the risk of negative and pessimistic emotional states (14), which may lead to sleep quality problems, such as difficulty falling asleep. Therefore, Hypothesis 3 was proposed: psychological stress level plays a moderating role between electronic device use and sleep quality.

In summary, existing studies have explored electronic device use, self-rated health, sleep quality, psychological stress, and other related factors among college students and other populations; however, comprehensive research elucidating the interrelationships among these four variables, particularly in medical students, remains limited. Drawing on the KAP theory and the biopsychosocial medical model, this study constructed a moderated mediation model to elucidate in depth the impact and underling mechanisms of electronic device use time on SRH among medical students, with sleep quality as a mediator and psychological stress as a moderator. The findings are expected to enhance medical students’ health awareness and self-perceptions of health across multiple dimensions, thereby promoting the advancement of the Healthy China Initiative.

## 2 Methods

### 2.1 Participants

In this study, convenience sampling was adopted to recruit medical students from universities in Hefei City, Anhui Province, as the main research participants. The survey was conducted using a combination of online and offline methods, with a total of 699 questionnaires collected. Questionnaires with excessive missing values were excluded, resulting in 679 valid questionnaires, with a valid response rate of 97%. Detailed information is presented in Table 1.

**Table 1:** Basic information on survey respondents (*n* = 679).

Variable	Item	*n*	Proportion (%)
Gender	Male	310	45.7
	Female	369	54.3
Age	Younger age group (15–19 years)	294	43.3
	Middle age group (20–24 years)	339	49.9
	Older age group (25–30 years)	46	6.8
School	Medical institution	630	92.8
	Non-medical institution	49	7.2
Grade	Lower grade group (Year 3 and below)	283	41.7
	Upper grade group (Year 4 and postgraduate)	396	58.3
Monthly living expenses	<1,000	28	4.1
	1,000–1,500	274	40.4
	1,501–2,000	280	41.2
	2,001–2,500	66	9.7
	2,501–3,000	15	2.2
	>3,000	16	2.4
Place of residence	Provincial capital (including municipalities)	97	14.3
	Prefecture-level city	145	21.4
	County seat	202	29.7
	Town/Village	235	34.6
Highest parental education level	Master’s degree or above	16	2.4
	Bachelor’s/Associate degree	131	19.3
	High school/Vocational school	175	25.8
	Junior high school	281	41.4
	Primary school or below	76	11.2
Number of chronic diseases in the family	None	516	76
	One	122	18
	Two or above	41	6
Regular physical examinations	Yes	129	19
	No	550	81

### 2.2 Measures

#### 2.2.1 Electronic device use time

An open-ended questionnaire was used to measure electronic device use time. Participants were asked to recall their average daily use time of electronic devices over the past month, including mobile phones, computers, tablets, smartwatches, and televisions. E-device use time was assessed by the question, “How long do you usually use electronic devices every day?,” rated on a 5-point Likert scale with the following response options: “less than 1 h,” “1–3 h,” “3–5 h (excluding 3 h),” “5–8 h (excluding 5 h),” and “more than 8 h.” Response were assigned scores from 1 to 5, respectively.

#### 2.2.2 Sleep quality scale

Sleep quality is defined as an individual’s self-satisfaction with all aspects of the sleep experience, which can be measured by the following variables: sleep efficiency, sleep latency, wake after sleep onset (WASO), and sleep architecture measurements (15). The Pittsburgh Sleep Quality Index (PSQI) was referenced to evaluate the sleep quality of medical students. As one of the most commonly used tools for assessing sleep quality in both non-clinical and clinical populations (16), the sleep quality scale in this study included 14 items, covering subjective sleep quality, sleep latency, sleep efficiency, sleep disturbances, hypnotic medication use, and other related contents. Subjective sleep quality was assessed using a 5-point Likert scale (“very good,” “good,” “fair,” “poor,” “very poor”). All other items were rated on a 4-point frequency scale, with response options of “none,” “<1 time/week,” “1–2 times/week,” and “≥3 times/week,” or “none,” “occasionally,” “sometimes,” and “often”. The total score ranged from 14 to 57, with the sum of all items representing the PSQI score. The scale was reverse coded, such that higher scores indicated poorer sleep quality. The Cronbach’s α coefficient of this scale in this study was 0.845.

#### 2.2.3 Self-rated health scale

Self-rated health refers to an individual’s self-assessed overall health status, which is widely used as a key health indicator for individuals of all age groups (17). The Short Form-36 Health Survey (SF-36) is one of the most commonly used tools for measuring health-related quality of life (HRQoL) in clinical practice and population studies (18). Self-rated health among medical students was assessed using SF-36, based on the second Chinese version translated and revised by Lu et al. (19). The self-rated health scale in this study included 17 items, specifically covering overall physical and mental health self-assessment (5-point Likert scale), restrictions on social and practical activities due to health (3-point Likert scale), current health status compared with the past (5-point Likert scale), and health status compared with others (5-point Likert scale). The sum of all item scores was calculated as the self-rated health score, with a score range of 17–50. The self-rated health scale was reverse coded, so that higher scores indicated poorer self-rated health. The Cronbach’s *α* coefficient of this scale in this study was 0.722.

#### 2.2.4 Psychological stress scale

The psychological stress scale for medical students was developed with reference to the Short Form-36 Health Survey (SF-36) and the Chinese College Student Mental Health Scale (CCSMHS). Participants were asked to report the frequency of stress-related feelings and emotional experiences over the past month (20). The scale included nine items (e.g., feeling tense, depressed, discouraged, very tired, and fatigued), each rated on a 5-point Likert scale: “1 = none of the time,” “2 = a little of the time,” “3 = some of the time,” “4 = most of the time,” and “5 = all of the time.” The sum of all item scores was calculated, with a score range of 9–45. This scale was scored in a positive direction, with higher scores indicating higher levels of psychological stress. The Cronbach’s *α* coefficient of this scale in this study was 0.876.

### 2.3 Statistical analysis

Data processing and statistical analyses were performed using SPSS version 26.0 and Microsoft Excel. The Harman’s single-factor test was employed to examine common method bias (CMB). Descriptive statistical analyses of each variable were conducted using mean and standard deviation, while Pearson correlation analysis was used to explore the correlations among variables. The moderated mediation effect analysis was implemented using Model 7 of the PROCESS macro developed by Hayes (21), based on the bootstrap method. Meanwhile, the moderator variable was divided into high and low groups based on ±1 standard deviation for the simple slope test. The significance level was set at *α* = 0.05.

## 3 Results

### 3.1 Baseline characteristics of the study subjects

In this study, the 679 medical students exhibited relatively balanced proportions distribution in terms of gender, grade level, and family residential tier. The age range was 15–30 years, with 93.2% aged 15–24. Monthly living expenses generally ranged between 1,000 and 2,500 yuan, accounting for 91.3% of the sample. The highest educational attainment of most parents was high school or secondary vocational education or below. Seventy-six percent of medical students reported no family history of chronic diseases or were unaware of such history, while 81% lacked regular health check-up habits. Details are presented in Table 1.

### 3.2 Common method bias test

Common Method Bias refers to the systematic error that is due to the selection of survey samples, the consistency of the test environment, and other interference factors in the process of investigation. Common method variance was analyzed using Harman’s single-factor test (22), and unrotated exploratory factor analysis was conducted on the raw items of the electronic device use time, sleep quality, self-rated health, and psychological stress scales. The analysis demonstrates that all 11 factors had the eigenvalues greater than 1. The first factor explains 23.67% of the variance, which is below the standard critical threshold of 40%. Therefore, no severe common method bias exists in this study.

### 3.3 Correlation analysis of variables

Pearson correlation analysis revealed that the average daily electronic device use time exhibited positive correlation with the scale scores of self-rated health, sleep quality, and psychological stress among medical students. Similarly, the sleep quality scale score showed positive correlation with the scale scores of self-rated health and psychological stress. Given that both sleep quality and self-rated health were reverse coded, the results indicate that electronic device use time was negatively associated with self-rated health and sleep quality, while being positively correlated with psychological stress. Moreover, sleep quality was positively correlated with self-rated health but negatively associated with psychological stress. Among the demographic variables, school, grade, monthly living expenses, family residence location, parents’ highest educational attainment, number of chronic illnesses in the family, and regular physical examination habits were associated with several key study variables. Details are shown in Table 2.

**Table 2:** Results of correlation analysis for each variable.

	Daily electronic device use time	Self-rated health	Sleep quality	Psychological stress	Gender	Age	School	Grade	Monthly living expenses	Place of residence	Highest parental education level	Number of chronic diseases in the family	Regular physical examinations
Daily electronic device use time	1												
Self-rated health	0.158**	1											
Sleep quality	0.221**	0.169**	1										
Psychological stress	0.06	−0.006	0.372**	1									
Gender	−0.039	0.026	0.018	0.034	1								
Age	−0.057	0.015	0.009	0.025	0.009	1							
School	0.025	0.057	−0.024	0.120**	−0.019	−0.001	1						
Grade	−0.047	0.013	0.06	0.065	0.083*	0.708**	0.051	1					
Monthly living expenses	0.05	0.063	−0.01	−0.120**	−0.100**	0.021	0.057	−0.005	1				
Place of residence	0.01	−0.009	0.012	0.01	0.013	0.03	−0.138**	0.002	−0.233**	1			
Highest parental education level	0.098*	0.009	0.084*	0.085*	−0.017	0.067	−0.02	0.041	−0.243**	0.355**	1		
Number of chronic diseases in the family	0.003	0.018	0.114**	0.033	0.083*	0.116**	0.023	0.125**	−0.022	0.022	0.067	1	
Regular physical examinations	0.126**	0.071	0.096*	0.100**	0.076*	−0.025	−0.039	0.017	−0.133**	0.119**	0.190**	0.018	1

Note: **p < 0.01,*p < 0.05. Columns 1–13 correspond sequentially to the row headings: “Daily electronic device use time”, “Self-rated health”,”Sleep quality”,”Psychological stress”,”Gender”,”Age”, “School”,”Grade”,”Monthly living expenses”,”Place of residence”,”Highest parental education level”,”Number of chronic diseases in the family”, and “Regular physical examinations”.

### 3.4 Mediation analysis of the effect of e-device use time on self-rated health through sleep quality among medical students

This study controlled for socio-demographic and household characteristics, using medical students’ e-device use time as the independent variable, sleep quality as the mediating variable, and self-rated health as the dependent variable. The Model 4 of the SPSS PROCESS macro was employed to analyze the data, and all variables are centered. The results demonstrated that the electronic device use time exhibited a positive predictive effect on the scale score of self-rated health (reverse-coded) (*β* = 0.149, *t* = 3.847, *p* < 0.001) and the scale score of sleep quality (reverse-coded) (*β* = 0.211, *t* = 5.565, *p* < 0.001). These findings indicate that electronic device use time was negatively associated with both sleep quality and self-rated health among medical students. After incorporating sleep quality as a mediator, the electronic device use time remained a positive predictor of the score of the self-rated health scale (*β* = 0.119, *t* = 3.035, *p* < 0.01), and the score of sleep quality scale positively predicted the scale score of self-rated health (*β* = 0.141, *t* = 3.611, *p* < 0.001). This suggests that the negative predictive effect of electronic device use time on self-rated health, and the positive predictive effect of sleep quality on self-rated health remained unchanged after introducing sleep quality as a mediator. Thus, sleep quality played a mediating role between electronic device use time and self-rated health. The 95% bootstrap confidence interval was [0.0591, 0.2217], which did not include 0, indicating a significant mediating effect that was partial. The mediating effect size was 0.181, accounting for 20.03% of the total effect (see Table 3).

**Table 3:** Regression analysis of the relationship of variables in the mandating model.

Regression equation		Goodness-of-fit indices			Coefficient significance	
Dependent variable	Independent variable	*R*	*R* ^2^	*F*	*β*	*t*
Sleep quality	Electronic device use time	0.273	0.075	5.377	0.211	5.565^***^
Self-rated health	Electronic device use time	0.190	0.036	2.509	0.149	3.847^***^
Self-rated health	Sleep quality	0.234	0.055	3.507	0.141	3.611^***^
Self-rated health	Electronic device use time				0.119	3.035^**^

Analyses controlled for all demographic and socioeconomic covariates. **p < 0.01, ***p < 0.001.

### 3.5 The moderating role of psychological stress in the mediating effect of sleep quality

Building upon the mediation effect, we further examined the moderating role of psychological stress. To test whether the first half of the mediation pathway was moderated by psychological stress, the PROCESS Model 7 was used to analyze the data. The results indicated that the electronic device use time was significantly positively correlated with the score of sleep quality scale (reverse-coded) (*β* = 1.2039, *t* = 5.5510, *p* < 0.001). The score of psychological stress scale (forward-coded) exhibited a significant positive predictive effect on the score of sleep quality scale (*β* = 0.4616, *t* = 10.3890, *p* < 0.001), suggesting that after introducing psychological stress as a moderator, the level of psychological stress was negatively associated with sleep quality among medical students. The interaction term between electronic device use time and psychological stress had a marginally significant predictive effect on sleep quality (*β* = 0.0727, *t* = 1.9230, *p* = 0.0550).

Johnson-Neyman significance region analysis revealed that the moderating effect of electronic device use time on sleep quality was not significant (*p* > 0.05) when psychological stress levels were below −7.3105 (accounting for 9.13% of the total sample); in contrast, this moderating effect became significant (*p* < 0.05) when psychological stress levels were above −7.3105 (accounting for 90.87% of the total sample). Additionally, the positive effect of electronic device use time on sleep quality scale score significantly positively predicted the self-rated health scale score (*β* = 0.7230, *t* = 3.0350, *p* < 0.01), indicating that the negative predictive effect of electronic device use time on sleep quality was significantly negatively associated with self-rated health among medical students. Collectively, these findings demonstrate that psychological stress moderated the first segment of the mediating pathway, and the moderated mediation effect was established (see Table 4).

**Table 4:** Mediation effects test with moderation.

Dependent variable	Equation 1 (sleep quality)			Equation 2 (self-rated health)		
	*β*	SE	*t*	*β*	SE	*t*
Electronic device use time	1.2039	0.2169	5.5510^***^	0.7230	0.2382	3.0350^**^
Psychological stress	0.4616	0.0444	10.3890^***^			
Electronic device use time × psychological stress	0.0727	0.0378	1.9230			
Sleep quality				0.1397	0.0387	3.6110^***^
*R*^2^	0.2040			0.0550		
*F*	14.2360^***^			3.5070^**^		

Analyses controlled for all demographic and socioeconomic covariates. **p < 0.01, ***p < 0.001.

**Figure 1:**
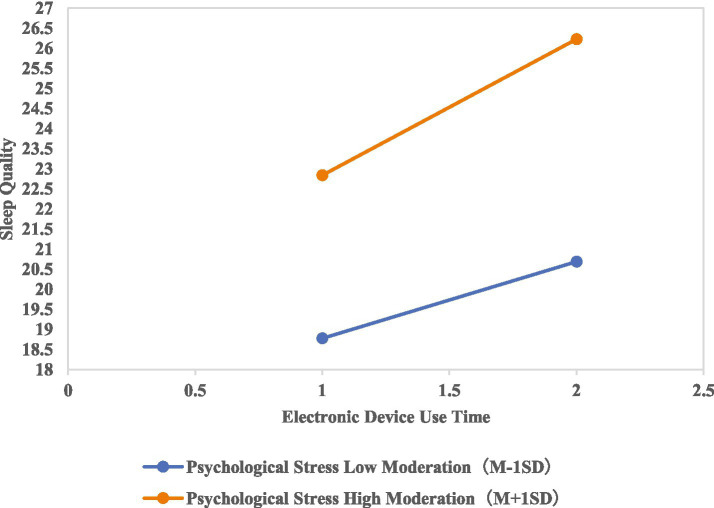
Moderating role of psychological stress on electronic device use time and sleep quality.

To further clarify the moderating strength of psychological stress and the robustness of its effects, a simple slope analysis was conducted to examine the impact of electronic device use time on sleep quality across different levels of psychological stress. The results showed that when psychological stress scale scores were at low (*β* = 0.8982, *t* = 3.1630, *p* = 0.0016), moderate (*β* = 1.2454, *t* = 5.8011, *p* < 0.001), and high levels (*β* = 1.5926, *t* = 5.3165, *p* < 0.001), psychological stress scale scores exhibited a significant positive predictive effect on sleep quality scale scores, with the positive predictive effect being stronger as psychological stress scale scores increased. Given that sleep quality was reverse coded in this study, this finding can be further interpreted as follows: psychological stress level among medical students was negatively associated with sleep quality, and the negative predictive effect strengthened as the levels of psychological stress increased. Furthermore, this conclusion was consistently verified by the Johnson-Neyman significance region analysis and the slope of the line in Figure 1.

## 4 Discussion

### 4.1 Electronic device use time negatively predicts self-rated health among medical students

Based on cross-sectional survey data, this study explored the influencing pathway of electronic device use time on self-rated health among medical students, providing empirical reference for the intervention of health behaviors and the improvement of health levels among medical students. The results demonstrated that electronic device use time significantly negatively predicted self-rated health among medical students, supporting Hypothesis 1.

This finding is consistent with the direction of conclusions from most existing studies. Previous research has confirmed the negative association between electronic device use and individual health: at the physiological level, excessive screen exposure is not only closely associated with decreased sleep quality, reduced physical activity, and increased sedentary time but may also exacerbate the risks of myopia, cardiometabolic diseases, neuroinflammation, and obesity (23). At the psychological level, prolonged screen time is prospectively associated with mental health symptoms in adolescents, increasing the risk of negative emotions (24), and exerting certain impacts on students’ cognitive abilities, language skills, and social–emotional adaptation. A meta-analysis further confirmed that longer screen time is associated with higher health risks (25). Additionally, studies have found that the type and purpose of electronic device use have significant impacts on adolescents’ mental health: the use of mobile phones and tablets is closely related to psychological issues such as academic stress and anxiety, while the use of electronic devices for chatting, gaming, and other purposes shows a stronger association with depression. Compared with ordinary college students, medical students face high-intensity academic pressure, clinical practice demands, and employment stress, making them prone to outbreaks of negative emotions and subsequent compensatory screen dependence, which may further increase health risks (26).

However, some studies have concluded differently from this study, suggesting that electronic device use can promote interpersonal communication among young people, facilitate access to health information, alleviate emotional stress, and enhance wellbeing (27). In reality, however, many young people may struggle to effectively translate acquired electronic health information into health-related behaviors due to immature cognitive development and limited self-regulatory capacity (28). Existing research has also confirmed that the overall prevalence of sub-health among medical students is 62.2%, and the use of mobile phones and computers may indirectly affect the physiological sub-health status of medical students by influencing their physical activity (5). Confronted with triple pressures from academics, practice, and employment, coupled with the frequent occurrence of tense doctor-patient relationships and public health incidents in recent years, medical students are more likely to experience poor sleep quality, depression, and other health problems (29). In this context, medical students may increase their electronic device use time (e.g., watching videos, searching for health information, learning more professional knowledge) to relieve stress, assist sleep, and even conduct health management. This does not conflict with the conclusions of this study; instead, it suggests that we should strengthen health education on health-promoting lifestyles for medical students, guide them to reasonably control nighttime electronic device use time, use electronic devices to obtain health information, and conduct regular health check-ups. Additionally, sports activities and mental health programs should be organized to alleviate the academic and employment stress of medical students, enhance their e-health literacy, and promote the translation of such literacy into practical actions.

### 4.2 The mediating role of sleep quality

Mediation effect analysis was employed in this study, which elucidated that sleep quality exerts a key mediating role between electronic device use time and self-rated health, supporting Hypothesis 2. This finding complements, to a certain extent, the gaps in existing research that have primarily focused on the mutual influence between electronic device use and sleep quality, as well as the impact of sleep quality on mental health. It also provides new empirical evidence for deciphering the mechanisms underlying the influence on medical students’ health.

The results of this study are consistent with the conclusions of most existing research. Previous studies have confirmed that electronic device use affects sleep quality through physiological and psychological mechanisms, among which blue light exposure inhibiting melatonin secretion is the core pathway (30). A study on adolescents showed that sleep problems are associated with anxiety, depression, increased heart rate, elevated blood pressure, and heightened stress (31), while positive health behaviors such as daily screen time less than 2 h, good sleep quality, and sufficient sleep duration are linked to favorable self-rated health (10). Based on the Media System Dependency Theory and the Time Displacement Hypothesis (32), medical students, under multiple pressures from practice and employment, tend to rely on electronic device use to meet their needs for entertainment, relaxation, and information acquisition, which in turn crowds out their time for study, physical activity, and rest. The pleasure derived from electronic device use may further extend its usage duration, potentially leading to sleep rhythm disruption. Sleep plays a crucial role in maintaining normal physiological functions, improving quality of life, and enhancing happiness, and it is one of the strongest predictors of self-rated health (33), as well as being associated with psychological issues such as anxiety among college students (34). Anderson’s Health Service Utilization Behavior Model also provides theoretical support for this study (35), indicating that electronic device use can directly affect physical and mental health or indirectly influence self-rated health through mediating pathways such as interfering with sleep behavior, cognitive function, and social interaction (36, 37).

However, certain discrepancies exist between the results of this study and those of some existing research. Some studies suggest that there is no significant association between electronic device use time and sleep quality, but the timing of mobile phone use before bedtime may affect sleep outcomes (38). Furthermore, different types of screen content consumed within 1 h before bedtime have varying impacts on sleep quality: activities such as watching movies, socializing, and browsing may reduce sleep quality, while sleep-aid videos may improve it (39). These discrepancies may stem from multiple factors: first, differences in measurement tools—this study used average daily electronic device use time as the independent variable, while some studies have focused on the timing or purpose of electronic device use; second, the particularity of the medical student group and potential cognitive biases may have influenced the results; third, the interference of reverse effects between variables, inadequate consideration of control variables, or the dilution of association strength by other potential influencing variables. For instance, some medical students may offset, to a certain extent, the negative impact of electronic device use on sleep quality through other health behaviors such as physical activity or diet; alternatively, the reverse effect of sleep quality on electronic device use time, as noted earlier, may also interfere with the results of this study.

Therefore, future research should subdivide the scenarios and purposes of electronic device use and incorporate more variables, such as diet and physical activity, for in-depth exploration. In practice, it is necessary to guide medical students to reduce electronic device use at night, implement multi-path interventions, including cognitive behavioral therapy, health behavior education, and monitoring, to help them develop good sleep habits. Medical students should also be encouraged to participate in physical activities, while strengthening employment training and psychological stress counseling to promote the development of healthy behaviors and the improvement of health awareness among medical students.

### 4.3 The moderating role of psychological stress

This study further confirmed that psychological stress moderated, to a certain extent, the predictive effect of electronic device use time on sleep quality among medical students. Specifically, higher levels of psychological stress were associated with a stronger negative predictive effect of electronic device use time on sleep quality, supporting Hypothesis 3. This finding is consistent with the “snowball effect model” (22), providing partial empirical support for deciphering the differential mechanisms underlying impaired sleep quality among medical students.

Existing studies have indicated that electronic device use time could affect adolescents’ sleep quality through psychological responses such as anxiety, stress, and mood disturbances (40, 41). Psychological stress refers to a set of specific psychological reactions that arise when individuals fail to effectively cope with pressures in study or work, typically manifested as anxiety, tension, fatigue, and withdrawal (42). Due to the particularities of their professional training system, clinical practice, and future employment, medical students face multiple current and future stressors, resulting in relatively high levels of psychological stress. Studies have shown that medical students or medical personnel are a high-risk group for psychological stress trauma; long-term exposure to stress is prone to inducing sub-healthy symptoms such as gastrointestinal disorders and sleep disturbances, which may impair physiological and psychological functions (43), consistent with the conclusions of this study.

By integrating the biopsychosocial (BPS) medical model (44) with the group-specific “dual academic-clinical pressure” experienced by medical students’, the moderating mechanism of psychological stress could be explained at multiple levels. At the physiological level, long-term psychological stress can continuously activate the hypothalamic–pituitary–adrenal (HPA) axis, disrupting the pre-sleep physiological rhythm of “decreased cortisol and increased melatonin.” Meanwhile, blue light from electronic devices can further inhibit melatonin secretion. Moreover, medical students often extend nighttime electronic device use time due to academic and practical needs, resulting in significantly longer average daily blue light exposure than ordinary college students (45). The combined effect of these two factors directly leads to the disappearance of the “sleep onset window,” consistent with recent research findings on high-stress populations. At the psychological and behavioral levels, based on the Cognitive Activation Theory and the Uses and Gratifications Theory, psychological stress may shift medical students’ motivation for electronic device use from “functional academic needs” to “escape-oriented stress relief,” thereby trapping them in a vicious cycle wherein electronic device use results in temporary stress relief, followed by stress recurrence and subsequent longer electronic device use. From the social dimension of the BPS model, medical students themselves face superimposed pressures of long electronic device use time, academic tasks, and clinical practice; under the stimulation of high psychological stress levels, their sleep quality may further decrease. This result is of great significance for stratified and classified interventions on medical students’ health behaviors.

This section explored the influencing mechanisms among electronic device use time, psychological stress, and sleep quality among medical students, verifying and expanding relevant theories. However, the moderating coefficient of psychological stress was relatively low, which may be attributed to the complex joint influence mechanism of the two factors on sleep quality: first, the dominant role of physiological mechanisms, such as melatonin inhibition caused by electronic device use, may be stronger; second, there may be a bidirectional relationship between psychological stress levels and sleep quality. Based on the “Compensation Theory,” medical students with high psychological stress levels may use electronic devices to relieve stress for short-term sleep assistance; third, poor sleep quality may inversely increase psychological stress levels or electronic device use time. All these factors may interfere with the estimation of the moderating effect. In the future, it will be necessary to incorporate professional health behavior measurement tools to collect more objective data on psychological stress levels and sleep, fully consider the bidirectional influence mechanisms among variables, and construct a more scientific and comprehensive model.

### 4.4 Study limitations

Despite the findings of this study, several limitations remain. First, constrained by its cross-sectional design, this study may not fully clarify the absolute causal relationship between electronic device use time and self-rated health among medical students. Additionally, self-administered questionnaires may introduce certain recall and self-report biases. Future research could employ professional health measurement tools and cohort studies to obtain more scientific longitudinal data. Moreover, this study was focused on one specific city, Hefei, Anhui Province, limiting its research scope. However, Hefei is a core hub for medical education in Anhui Province and ranks at an upper-middle level nationally in terms of socioeconomic development, making it broadly representative of such similar cities. Furthermore, given the common features of current medical training models and shared academic and clinical pressures, the findings of this study could be generalized to the broader medical student population. Finally, this study only examined the internal mechanisms involving four primary variables: electronic device use, sleep quality, self-rated health, and psychological stress. Future research could further explore the roles of device type, usage purposes, and time of use within various pathways, incorporate additional mediating and moderating variables (e.g., type and purpose of electronic device use, diet, physical activity), and consider more control variables such as contextual environmental factors, family influences, and policy factors to further enrich the understanding of self-rated health status among medical students.

## 5 Conclusion

In summary, this study innovatively investigated the relationships and mechanisms among electronic device use time, psychological stress, sleep quality, and self-rated health in medical students. The findings demonstrate that electronic device use time negatively predicts self-rated health in this population, with sleep quality exerting a mediating role and psychological stress moderating the first segment of the mediating pathway. In light of the above results, future research should further expand the sample scope and incorporate more variables for in-depth exploration. Based on cognitive behavioral therapy, efforts should be made to improve the self-rated health levels of medical students and promote their physical and mental health development through multiple aspects, including health knowledge and policy promotion, health behavior education, stress counseling, health behavior monitoring, regular health check-ups, and the translation of health knowledge into practical behaviors.

## Data Availability

The original contributions presented in the study are included in the article/supplementary material, further inquiries can be directed to the corresponding authors.
